# Decolourizing distillery spent wash using fungal biotechnologies: from pollution to potential

**DOI:** 10.1186/s40643-026-01021-8

**Published:** 2026-03-26

**Authors:** Anusha Priya Singh, Sayli Dongre, Shaifali Sharma, Kriti Joshi, Harsh Bagdare, Ragini Bobade, Om Prakash, Rohit Sharma

**Affiliations:** 1 National Centre for Microbial Resource (NCMR), National Centre for Cell Science (NCCS), NCCS Complex, Ganeshkhind, Pune, Maharashtra 411 007 India; 2 Department of Aquatic Microbial Ecology, Institute of Hydrobiology, Biology Center, Ceske Budejovice, Czechia; 3 Faculty of Science, University of South Bohemia, Ceske Budejovice, Czechia; 4 School of Modern Science, Scope Globel Skill University, Hoshangabad Road, Near Misrod, Bhopal, Madhya Pradesh 462026 India; 5 Symbiosis Centre for Climate Change and Sustainability (SCCCS), Symbiosis International (Deemed University), Lavale, Pune, Maharashtra 412 115 India; 6 Department of Biotechnology and Microbiology, Sardar Vallabhbhai Patel College, Madleshwar, Madhya Pradesh 451 221 India; 7 Department of Microbiology, S.P. Pune University, Pune, Maharashtra 411 007 India; 8 School of Sciences, Sanjeev Agrawal Global Educational University, Bhopal, Madhya Pradesh 462 043 India

**Keywords:** Decolourization, Distillery spentwash, Effluent, Fungi, Molasses

## Abstract

Climate change and environmental pollution are among the most pressing global challenges today, with water pollution standing out as a particularly critical issue. Industrial wastewater discharge, especially from distilleries, significantly contributes to the degradation of aquatic and terrestrial ecosystems. Molasses-based distilleries are major perpetrators, producing vast quantities of dark brown effluent known as spent wash. This colouration is largely due to the presence of melanoidin, a recalcitrant compound formed via the Maillard reaction. Although many distilleries now utilize anaerobic digestion to convert this organic-rich waste into biogas, the resultant biomethanated spent wash remains highly coloured and environmentally hazardous. Direct discharge of untreated or partially treated spent wash into rivers, lakes, or soil severely disrupts ecological balance and poses risks to biodiversity. Existing disposal practices, such as lagoon storage or composting with press mud, offer limited solutions to the colour problem. Fungi, particularly those producing laccase and other oxidative enzymes, have demonstrated promising potential for decolourizing spent wash in laboratory studies. However, the enzymatic pathways involved in melanoidin degradation are still not fully understood. To address the persistant colour challenge, integrated treatment strategies combining fungal systems with complementary physical or chemical processes (eg, adsorption or advanced oxidation) may be required to achieve effective decolourisation. Such advancements are vital for creating effective, eco-friendly solutions to mitigate the environmental impact of the distillery industry and promote a circular bioeconomy.

**Graphical abstract:**

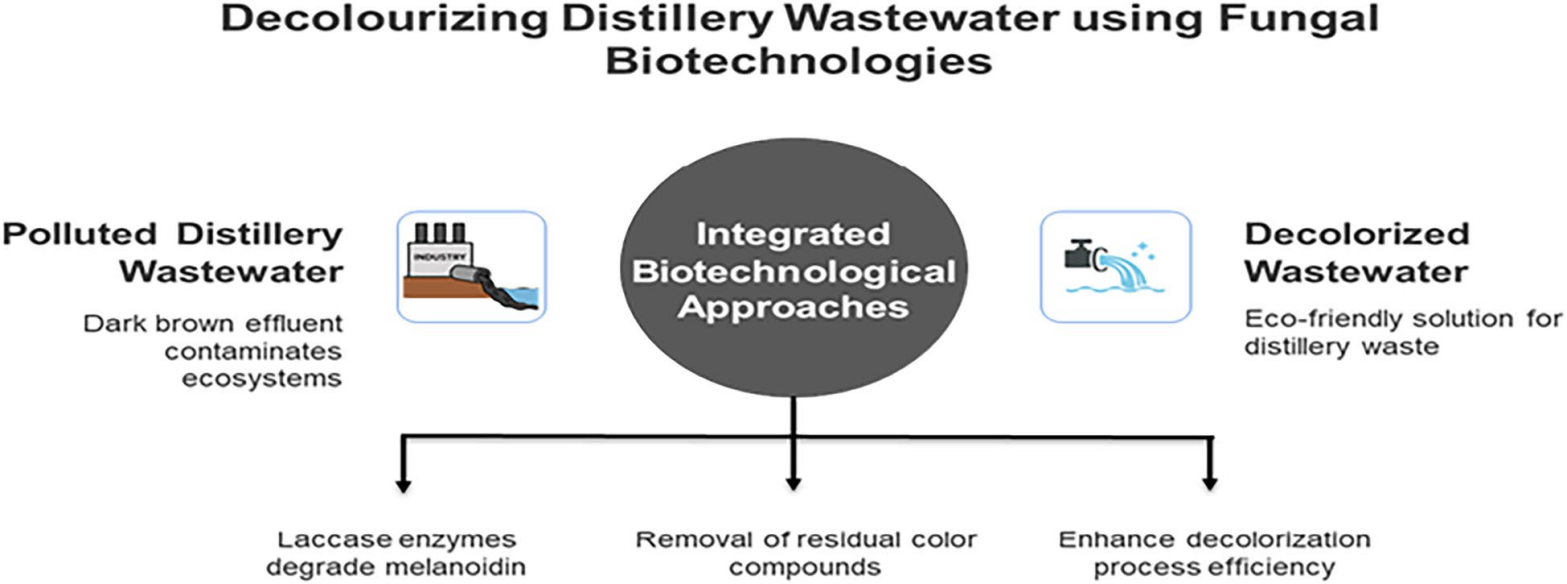

## Introduction

Water is a necessity for human survival, and access to clean water is a basic right. In the ancient world, most cities were established on riverbanks because of the need for water for irrigation, drinking, transportation, and livelihood. The advent of the Industrial Revolution in the eighteenth century raised the problem of wastewater generation and its subsequent treatment (Angelakis et al. 2022). This has reduced the availability of clean water for the human population and cattle, and has created a need for wastewater treatment to be reused at a global scale. Industrial pollution has primarily affected rivers and seas worldwide. According to Sanders et al. (2024), 48.8% of the world’s native freshwater mammals and 33% of its native freshwater amphibians are at risk of extinction. Moreover, in most rivers, harbors, and dams, natural cleaning does not occur. Environmentalists and governments are now working to provide clean water and sustainable development (Umair Hassan et al. 2021; Hoarau et al. 2018; Jeebon et al. 2025). Countries across the world have initiated efforts to clean municipal and industrial pollutants from the rivers. Industrial pollution is considered the most significant pollution sector, and considerable attention is being given to treating industrial pollutants (Kato and Kansha 2024). Major industries that cause severe pollution are paper and pulp, iron and steel, mines and quarries, food industry, brewing, dairy, organic chemicals, textiles, energy, and distillery industries (Ashrafi et al. 2015). Molasses (sugarbeet and sugarcane) based alcohol distilleries are recognized as red-zone industries in many countries and discharge huge amounts of untreated spent wash into natural reservoirs (Kumar and Chopra 2012).

**Fig. 1:**
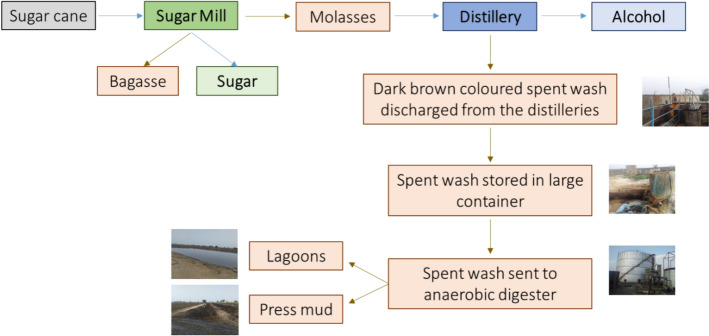
Schematic diagram of a molasses-based distillery process outlining the major steps involved in alcohol production from molasses, including fermentation, distillation, and the generation of the distillery spent wash as one of the by-products

Alcohol is generally produced in three main forms: rectified spirit, which is useful for industrial applications; extra neutral alcohol, which is used in the manufacturing of potable liquor; and fuel ethanol, or absolute alcohol, which is employed in blending with petrol. The demand for ethanol is rising due to biofuel policies in various countries that require fuel to be blended with 5–20% ethanol (Lundberg et al. 2023). The manufacturing process of alcohol involves three key steps: fermentation, distillation, and effluent treatment and disposal (Fig. 1) (Walker 2011). In distilleries, sucrose and reducing sugars in molasses are converted into ethyl alcohol and carbon dioxide by yeast enzymes. It is estimated that one million tons of molasses can yield approximately 200–290 L of rectified spirit through fermentation (Inamdar 1994). The distillation process separates alcohol from the fermented wash and concentrates it to 95–96% purity (v/v). This is achieved using either an atmospheric pressure distillation system or a multi-pressure system. Distilleries require a significant amount of freshwater for various processes, including diluting the spirit during distillation, generating steam, and providing makeup water for cooling towers. On average, producing one liter of alcohol requires about 10–17 L of water (Wu et al. 2009; Madaleno et al. 2020).

## Methodology

This review was prepared following a comprehensive literature search using major databases, including Scopus, Web of Science, and PubMed. The search was conducted using combinations of relevant keywords: “*distillery spent wash*”, “*molasses wastewater*”, “*effluent*”, “*fungal decolourisation*”, “*melanoidin*”, and “*bioremediation*”. Peer-reviewed articles published up to 2025 were considered. Studies focusing on non-biological treatment methods, wastewater streams unrelated to distillery or molasses-based industry, as well as conference abstracts and editorials, were not included. In addition to the literature survey, the authors conducted field visits to some distilleries and effluent release sites in Maharashtra, India, and Western Ghats forests to gain practical exposure to distillery effluent management practices and associated impacts. Observations from these visits were used to support and conceptualise the findings reported in the manuscript.

## Problem of spent wash (Effluent)

**Fig. 2:**
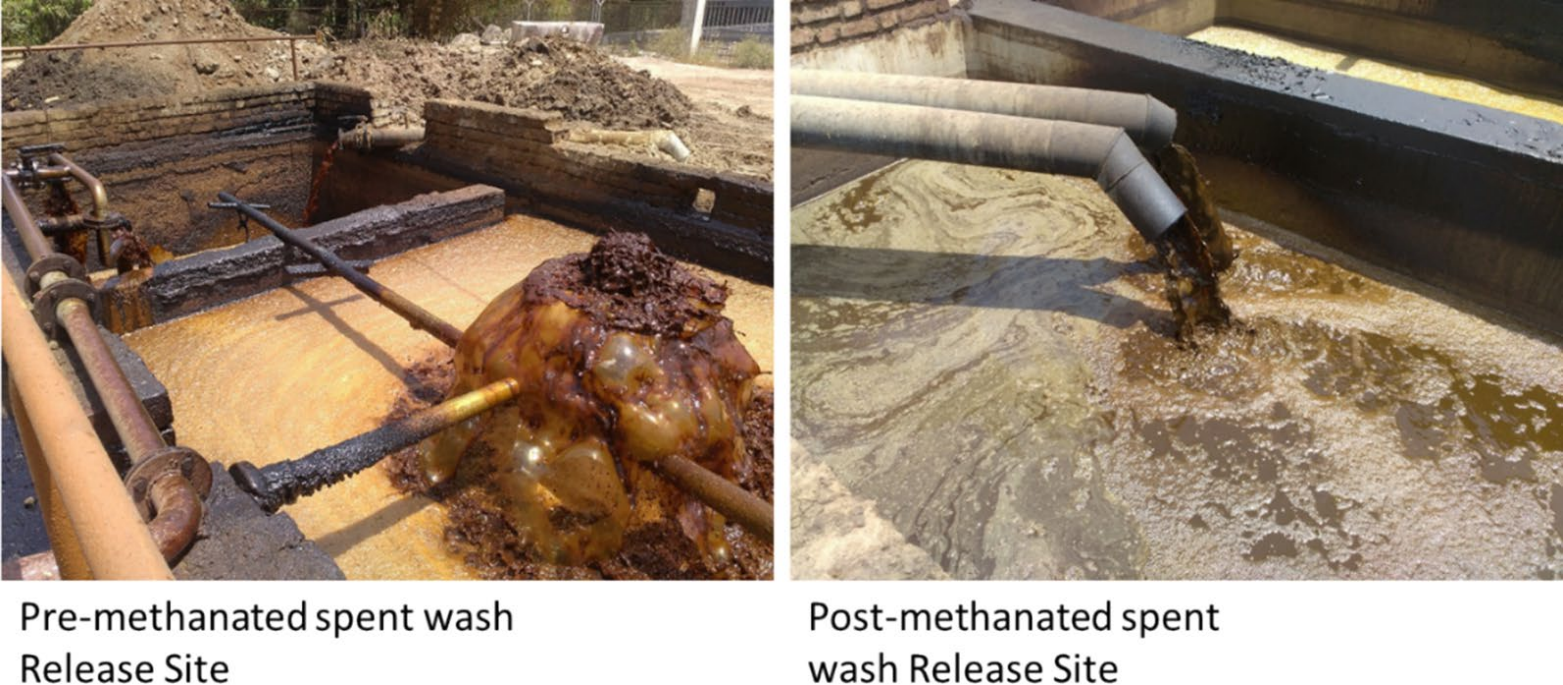
Release sites of spent wash, before and after biomethanation treatment from the state of Maharashtra, India. The photographs depict **a** an untreated spent wash discharge site, and **b** a post-methanated spent wash release site, demonstrating visual differences in colour intensity, turbidity, temperature, and environmental impact due to biomethanation

**Fig. 3:**
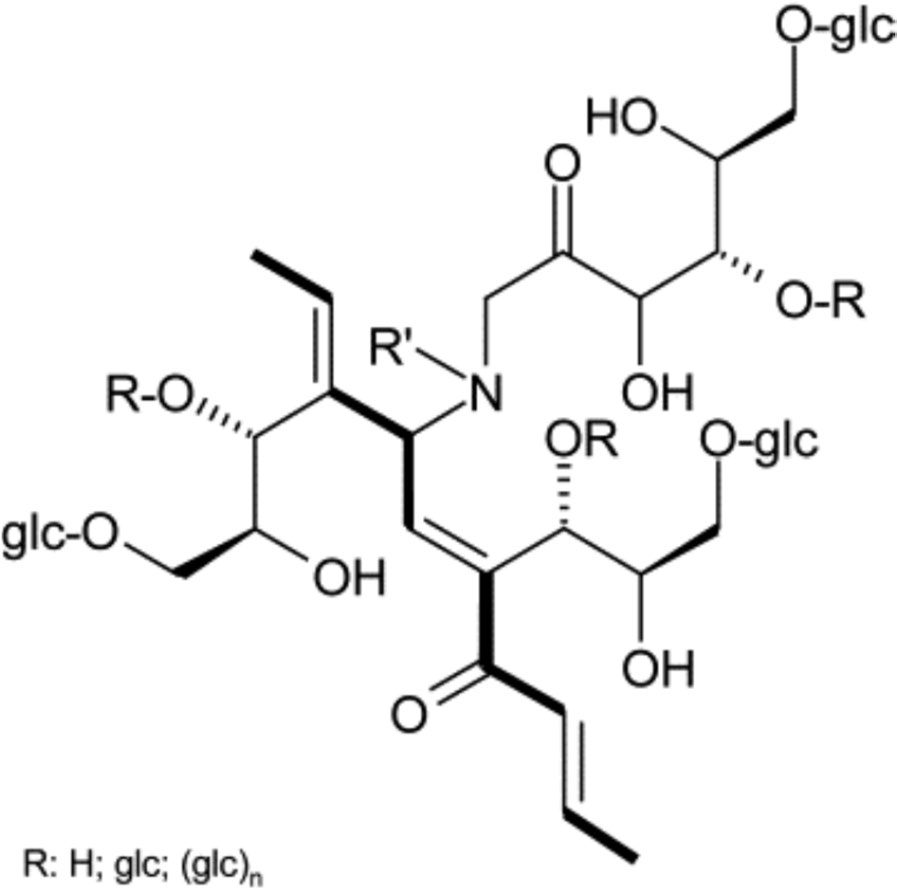
A representative structure of a glucose-glycine melanoidin formed through the Maillard reaction. Melanoidin is a polymeric, high-molecular-weight brown pigment generated by non-enzymatic condensation between reducing sugars and amino acids, highlighting its heterogeneous and complex nature. Source: Rufián-Henares and Morales 2011

**Fig. 4:**
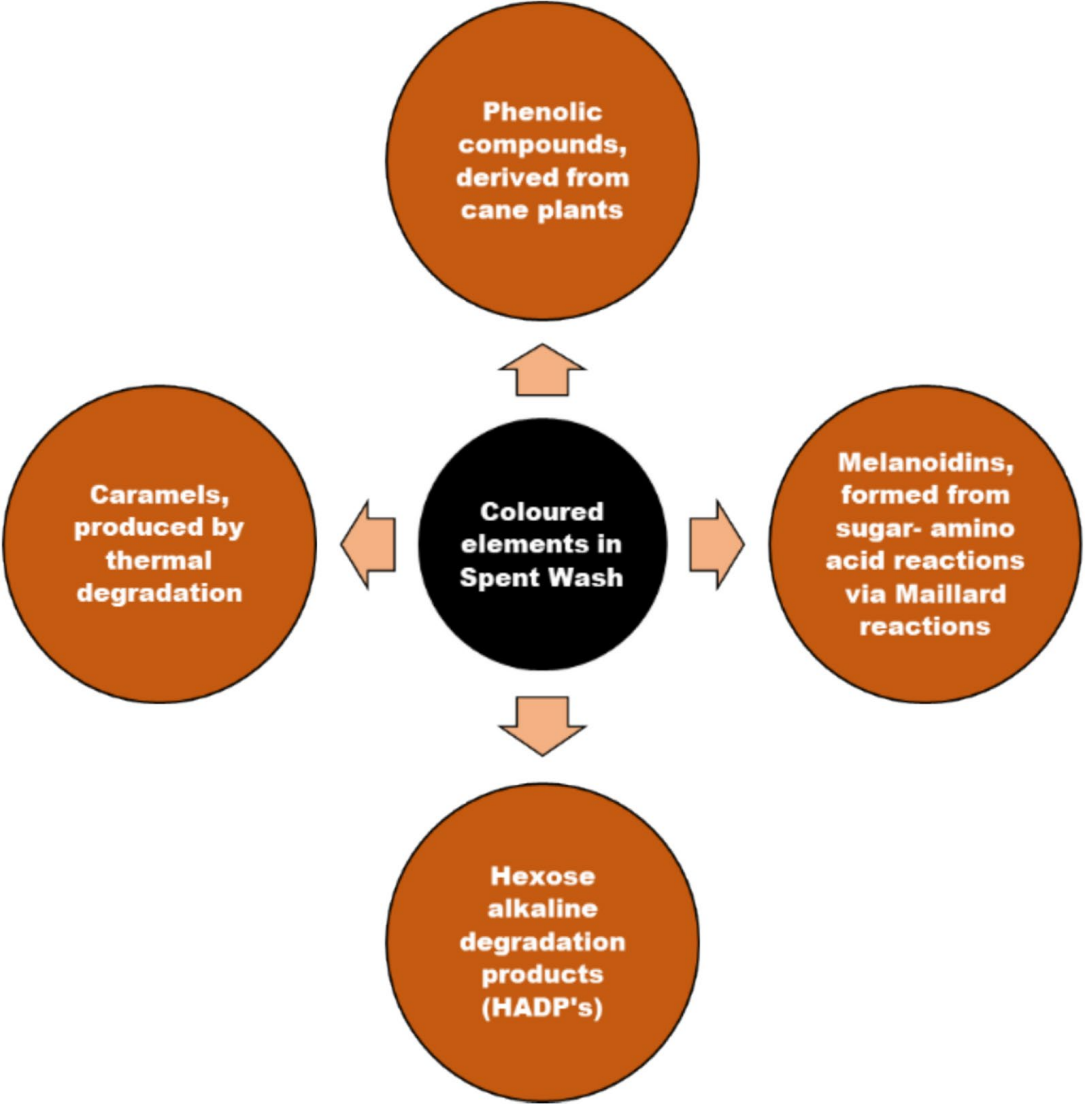
Coloured constituents present in the distillery spent wash. The figure highlights the major colour-imparting components of spent wash, primarily melanoidins, along with other organic compounds formed during the Maillard reaction and the distillery’s thermal processing

Distilleries use large amounts of water to function, which is released at the end as a spent wash (effluent), with high biological and chemical oxygen demands (BOD, COD) and high amounts of inorganic substances like chlorides, sulfates, phosphates, sodium, and potassium (Patel 2018). It also has coloured compounds like melanoidins, caramel, polyphenols, and an unpleasant odor. (Pant and Adholeya 2007a; Kharayat 2012). Biomethanation of spent wash produces large amounts of methane and is used to meet the energy requirements of most sugar mills associated with these distilleries. The biomethanated spent wash has lower BOD and COD (compared to raw spent wash), but the organic load is still high, and the unpleasant odor and dark brown colour remain unchanged (Mohana et al. 2009). The brown colour of spent wash is due to a complex mixture of colourants like melanoidin, caramels, phenolics, and other xenobiotic compounds (Pandey et al. 2003) (Figs. 2 and 3), and the obnoxious odor due to ketole, indole, and other sulfur compounds (Sharma et al. 2007; Chandra et al. 2008). Melanoidins, the major contributor of colour, are acidic, brown polymers consisting of negatively charged colloids due to carboxylic acids and phenolic groups (Manisankar et al. 2004). However, these are also natural polymers produced during browning reactions between amino acids and carbonyl groups at > 50 °C and low pH (Maillard reactions). The spent wash contains 2% of a dark brown melanoidin pigment with the empirical formula C17-18H26-27O10N and a molecular weight of 5–40 kDa (Fig. 4).

Melanoidins are high-molecular-weight, heterogeneous polymers formed via Maillard reactions between reducing sugars and amino compounds during fermentation and evaporation of molasses (Ali et al. 2024). The aromatic, nitrogen-containing, cross-linked structure and metal-binding ability make it poorly biodegradable and resistant to normal biological treatment (Bhosale et al. 2025). General physico-chemical methods do not fully address its degradation. Some microbes show promising results in their breakdown and could be a good source for developing an integrated spentwash treatment technology.

These compounds are recalcitrant and escape wastewater treatment processes, thereby entering the environment. The spent wash, when released in the environment, causes eutrophication in the water bodies, thus hindering sunlight penetration, reducing photosynthesis, and oxygen demand, affecting the aquatic life. Several researchers have studied the impact of the spent wash on aquatic life (Saxena and Chauhan 2003; Matkar and Gangotri 2003; Kumar and Gopal 2001; Ramakritinan et al. 2005). The studies reported behavioral changes, hematological alterations, changes in oxygen consumption, organ-level toxicity, and damaged respiratory processes in aquatic animals due to spent wash. Even at low concentrations, disposal of spent wash can alter the soil’s chemical composition, causing reduction of soil alkalinity and availability of micronutrients like manganese, adversely affecting seed germination and plant growth (Kannan and Upreti 2008). The spent wash also causes the discharge of protein and carbohydrates from seeds and reduces enzymatic activities (alkaline phosphatase and ATPase) (Mohana et al. 2009). Through seepage, it also affects colour, pH, electrical conductivity, and other physico-chemical properties of soil and groundwater (Jain et al. 2002). However, the extent of the deleterious effect on soil properties and microbial health depends on the duration of spent wash usage, affecting the ecosystem, soil fertility, and crop productivity.

Being rich in organic matter, it has high chemical oxygen demand (COD) and biological oxygen demand (BOD), which act as a major source of terrestrial and aquatic pollution (Tripathi et al. 2022). As per the analysis we conducted on our samples from Maharashtra sites, BOD for the pre-biomethanated spent wash ranged from 68,000 to 80,000 mg/L, and the spent wash from 34,000 mg/L. Physicochemically, it was dark in colour, low in pH, high in phenolics, organo-metallics, and heavy metals (Bhosale et al. 2025). There are several reports of the harmful effects on the aquatic ecosystems, like lagoons. It suppresses photosynthesis, depletes oxygen, and drives eutrophication, impacting aquatic flora and fauna (Bhosale et al. 2025). The toxic nature of melanoidin is well known in terrestrial ecosystems and also reported in cattle. Even anaerobically treated/biomethanated distillery spent wash (DSW) and incinerated ash retain toxic organometallic and carcinogenic compounds that harm fish, crops, and soil microbiota.

## Current storage and treatment strategy for spent wash

### Storage in lagoons

**Fig. 5:**
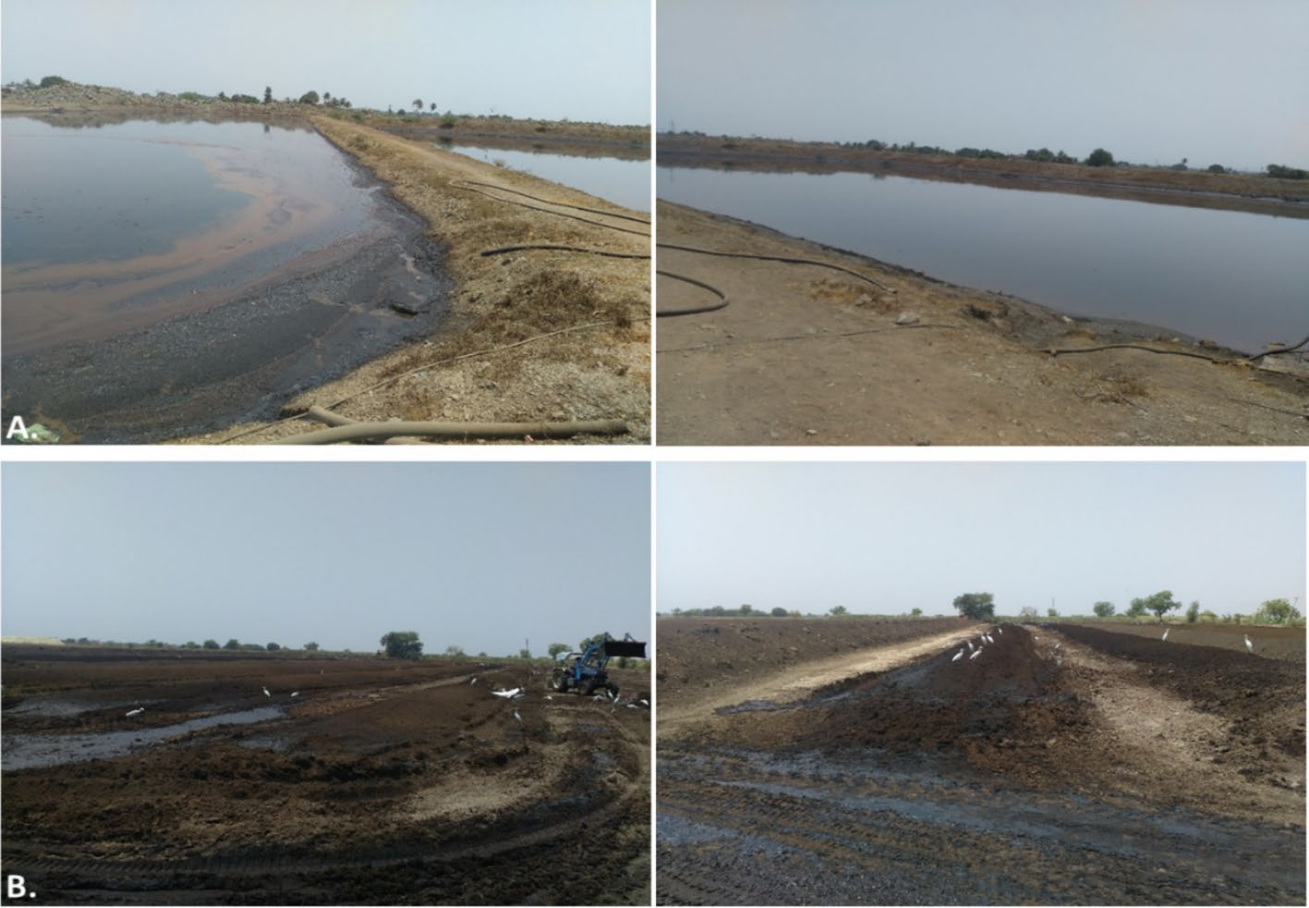
Actual site photographs showing storage and partial treatment of biomethanated spent wash. The above photographs illustrate **a** lagoon-based storage of biomethanated spent wash, and **b** partial treatment through press mud composting, demonstrating commonly adopted waste management practices at distillery sites

Most distilleries have opted to store spent wash in lagoons, which are large rectangular storage tanks. 70–100 m × 25–35 m × 3–5 m (l × w × d) (Fig. 5). These are not scientifically designed and allow spent wash to leach into the soil through seepage, thereby polluting soil and groundwater (Umair Hassan et al. 2021). The ideal way to prepare a lagoon is to make it impermeable by cementing, which prevents spent wash from seeping into the soil and/or underground water. When the authors visited one of the distilleries in Maharashtra, India, they observed that spent wash was polluting the environment due to leaching from the lagoons. Storage in lagoons does not help to reduce the colour or pollutants from spent wash (Ruhela et al. 2020). It is just a way to store and supply to the farmers to be used as fertilizers or used for spraying on press mud composting units (Fig. 5). In addition, it allows the coloured compound of spent wash, mainly melanoidin, to reach the agricultural fields, polluting a much larger area.

### Methanation

Anaerobic digestion for bio-methanation of spent wash is one of the treatments followed by most distilleries (Mohana et al. 2009). Since spent wash is rich in nutrients, it supports the growth of anaerobic bacteria and archaea, leading to biogas production. The biogas is converted into electricity, thereby meeting most of the distilleries’ energy requirements. The spent wash released by biomethanation is much darker in colour, and thus, there is no reduction in the brown colour and compounds responsible for the colour. Even though oxygen demand is reduced, it does not solve the problem of colour, a major pollution issue (Arimi et al. 2015). The biomethanated spent wash is released after diluting with freshwater either in the rivers (during monsoon) or sold as biofertilizers to farmers (Raghukumar et al. 2004; Kumar et al. 2007).

## Crop production using distillery spent wash

Pre-methane spent wash is rich in nutrients (calcium, magnesium, sulfate, iron, copper, sodium, and zinc), organic nitrogen, and phosphate. If diluted, it is used to irrigate crop fields, avoiding chemical fertilizers, and is sold to the farmers at a low cost (Umair Hassan et al. 2021). Distilleries combined with sugar industries give it under a buyback scheme wherein they purchase their sugarcane and give spent wash fertilizer at subsidized rates. Distillery spent wash has been used on several vegetable crops to increase the productivity and nutrient content of pulses, herbs, horticultural crops, vegetable plants, and creepers. (Chandraju et al. 2012; Chidankumar and Chandraju 2009; Chidankumar et al. 2010a, 2010b, Gahlot et al. 2011). A detailed list of crops, vegetables, and herbs, along with the effect of spent wash on them, is compiled in Table 1. It has a positive effect on the chemical composition of soil; it improves the organic carbon content, total N and P content, electrical conductivity, soil water retention, and penetration resistance (Hati et al. 2004). Experimental studies have shown that spent wash can enhance plant physiological performance, including plant growth, photosynthetic activity, transpiration rate, and stomatal conductance, primarily due to increased availability of macro and micro nutrients such as N, P, K, Ca, Mg, and trace elements in soil. Additionally, spent wash when tested along with additional inorganic NPK fertilizers for cereals has a positive effect on soil microflora and an increment in nutrient availability (Naveed et al. 2018; Kalaiselvi and Mahimairaja 2009). These improvements in soil fertility and plant processes collectively contribute to higher crop productivity, seed germination, root development, nutrient and water uptake by plants. Post-methanation spent wash significantly increased total organic carbon, total Kjeldahl nitrogen, potassium, and phosphorus content of soil, favoring seed germination and improved seedling growth of crops like pearl millet, sunflower, wheat, and sugarcane (Kaushik et al. 2005; Deshpande et al. 2012, 2017; Jayshree et al. 2022). Several researchers have tested the post-methanation spent wash in sugarcane and observed its positive effect on sucrose profile, mitotic efficiency, bud sprouting, root number and length, chlorophyll content, and catalase activity. It also induced harmful effects on humans, such as de novo chromosomal aberrations, like clump formation, chromosome stickiness, laggards, micronuclei formation, etc. (Selvamurugan et al. 2013; Kalaiselvi and Mahimairaja 2009; Jain and Srivastava 2012). Although a lot of published literature exists on the use of spent wash for irrigation, demonstrating its effectiveness in enhancing crop yields and nutrient content while simultaneously increasing soil fertility without depending on inorganic fertilizers, data on the long-term sustainability of distillery spent wash for irrigation and its effects on soil dynamics are limited (Rajkishore and Vignesh 2012). Whether it is stored or used for composting or crop production, the harmful effects of spent wash remain and are transferred to the soil and underground water. Colour removal of the spent wash has remained a challenge to experts in this field. Removal of colour either before or after biological treatment would help to reduce several problems in the final disposal of treated effluent. Hence, there is a need to develop biotechnology that is economical and provides a solution to the ever-increasing problem of pollution of water bodies by spent wash. In our view, fungi can provide this solution, and we discuss the reasons and scientific basis for our claim in the sections below.

**Table 1:** Detailed list of crops, vegetables, and herbs along with the effect of spent wash

S. no	Name	Comments	References
1	*Amaranthus gangeticus*	Improved nutrient quality when field irrigated with 33% spent wash	
	*Coriandum sativum*		
	*Trigonella foecum graceum*		
	*Peucedanum graveloens*		
	*Spinacia oleraceae*		
2	*Phaseolus mungo*	Improved nutrient quality when field irrigated with 33% spent wash	Chandraju et al. (2008)
	*Vigna catjang*		
	*Dolicos lablab*		
	*Cajanous cajan*		
3	Vegetables	Nutrient uptake by crops when the soil was pre-treated with spent wash	Chidankumar and Chandraju (2009)
4	*Lagaeria vulgaris*	Improved nutrient quality when field irrigated with 33% spent wash	Chidankumar et al. (2010a, b)
	*Benincasa hipsida*		
	*Cucurbita maxima*		
	*Trichosanthes anguina*		
	*Luffa acutangula*		
	*Momordica charantia*		
5	*Lycopersicon esculentum*	Better seed germination in all crops (except tomato) was observed	Ramana et al. (2002)
	*Capsicum annuum*		
	*Lagenaria siceraria*		
	*Cucumis sativa*		
	*Allium sepa*		
6	*Allium sativum*	Increased yield upon irrigation with 33% and 50% spent wash	Chidankumar and Chandraju (2009)
	*Zinziber officinale*		
	*Curcuma domestica*		
7	*Ocimum sanctum*	enhanced yield with better nutritional qualities with 33% spent wash irrigation	Chandraju et al. (2010, 2015)
	*Ocimum basilicum*		
	*Lucas aspera*		
	*Plectranthus amboinicus*		
8	Aster	A 1:3 dilution of spent wash increased flower production yield, whereas a 1:1 dilution killed the plants	Chandraju et al. (2013)
	Daisies		
	Jasmine		Chandraju et al. (2016a, b, c)
9	*Cicer arietinum*	Higher concentrations of spent wash resulted in delayed seed germination and poor seedling growth. However, post-harvest soil had an increased nutritional value	Gahlot et al. (2011)
10	*Oryza sativa*	Plant growth and photosynthesis increased at 5% spent wash concentration	Naveed et al. (2018)
11	*Oryza sativa*	Higher grain availability and increased micronutrient quality of soil were recorded with (%) X diluted spent wash	
12	*Oryza sativa*	Increased grain and straw yield when NPK fertilizer was used in addition to spent wash. Also improved mineral recycling and soil microflora	Kumar et al. (2012a)
	*Triticum aestivum*		
13	*Pennisetum galucum*	50% post-methanated effluent favoured seed germination	Kaushik et al. (2005)
14	*Helianthus annuus*	Sustainable yield and improved sodic soil	Deshpande et al. (2012)
15	*Saccharum officinarum*	Better development, growth, and yield of the crop, with improved mitotic activity, bud sprouting, and chlorophyll content. Higher doses (100 mL/kg) were detrimental to the crop	Rath et al. (2011)
			Jain and Srivastava (2012)
			Srivastava and Jain (2010)
16	*Saccharum officinarum*	Increased yield of sugarcane and an increase in exchangeable Ca, Na, Mg, and K in soil with spent wash and biocompost	Jayashree et al. (2022)
17	*Aracis hypogea*	Improved soil enzyme activity and nutrient replenishment in the crop cycle with split doses of spent wash	Kalaiselvi and Mahimairaja (2009)
18	*Amorphophallus paeonii folius*	Increased root and shoot length and increased yield of the plant; increased carbohydrate, beta carotene, and Vitamin C content	Jayashree et al. (2022)
19	*Vigna radiata*	Treated distillery effluent is suitable for legume crops irrigation	
20	*Nerium Oleander*	The sprouting, growth, and yield of the plant were very good (100%) in 1:3 spent wash irrigation,	Chandraju et al. (2016a, b, c)

## Mycoremediation

Presently, biomethanation is the main part of the entire treatment process for spent wash. Although biomethanation reduces the BOD and COD of spent wash, the dark brown colour remains, making it unsuitable for discharge or re-use (Satyawali and Balakrishnan 2008; Bhosale et al. 2025; Prajapati and Chaudhari 2015; Patel 2022; Bhardwaj et al. 2019). Colour removal using various physical, chemical, bio-adsorption, and biodegradation methods has been reported, but none have been effectively commercialized at distillery sites. (Mohana et al. 2009). Hence, there is a need for developing an eco-friendly method for the treatment of spent wash. Bacteria and fungi (mycelial and yeast) have both been reported for the biodegradation of colouring compounds of spent wash (Sirianuntapiboon et al. 2004; Vasanth Kumar et al. 2006). Colour removal and melanoidin degradation up to 90% have been achieved on diluted spent wash in lab-scale experiments by either single microbial strains or in consortia. However, since fungi are known to produce extracellular laccase and degrade dyes and have shown better results in laboratory experiments, they have better potential to be used in the field. (Strong and Burgess 2008; Knapp et al. 1995).

### Types of fungal bioremediation

The bioremediation of melanoidin and other coloured compounds of spent wash by fungi can be done by three different processes: biosorption, biodegradation, and bioaccumulation. Biosorption is considered most advantageous for the treatment of coloured waters and is identified as the preferred technique for decolourization by giving the best results (Kaushik and Malik 2009; Kabbout and Taha 2014; Tiwari et al. 2014) the best results.

#### Bioabsorption

Although bioabsorption is fast and requires less interaction time between the spent wash and mycelial biomass, the amount of biomass required is huge. Unlike other microbes, fungi show better adsorption capacity due to the special structure of the mycelial mat. In addition, it also secretes extracellular enzymes for the breakdown of complex substrates (Patel and Jamaluddin 2018). This property of the biomass reduces the retention time of the spent wash by the distilleries. Once the melanoidin is absorbed, which usually takes a short time, the mycelial mat can be separated and left for degradation of the melanoidin. Once the coloured compound is broken down, the mycelial mat can be reused. After initial adsorption, the mycelial mat, now bound with melanoidin, can be isolated and subjected to further enzymatic degradation, leading to partial or complete decolourization. Notably, once the adsorbed melanoidin is broken down, the fungal biomass can be regenerated and reused, improving the sustainability and economic viability of the process.

#### Bioaccumulation

Some fungi can accumulate the colour compounds inside the mycelia, even though they do not degrade them. Practically, the process may not be viable for industrial biotechnology as it will require a lot of mycelial biomass to treat large volumes of spent wash (Alexander 1999). Certain fungal species are also capable of bioaccumulating colour compounds within their intracellular compartments. Unlike enzymatic degradation, this mechanism involves the uptake and storage of melanoidin or related pigments without necessarily breaking them down (Alexander 1999). While this method can contribute to decolourization, its practical application at an industrial scale is limited, as it would require large amounts of fungal biomass to treat high volumes of spent wash. Moreover, without active degradation, the accumulated compounds may persist in the biomass, leading to disposal challenges. Therefore, although bioaccumulation is biologically interesting, it is generally considered less viable than enzyme-mediated bioadsorption and biodegradation strategies in the context of industrial spent wash treatment.

#### Biodegradation

There is a gap in our knowledge of the ability of fungi to degrade the coloured compounds responsible for the colour of spent wash. There are many studies on the macrofungi (mostly wood-rotting fungi), microfungi, and yeasts for the removal of colour from spent wash. Moreover, there are specific studies that examine the degradation of melanoidin. These fungi can degrade the colour extracellularly, also by the release of laccase group enzymes. The laccase group of enzymes, which belong to the oxidoreductase family and include laccase, manganese peroxidase, lignin peroxidase, etc., can significantly decolourize melanoidin-containing effluents in lab-scale studies (Loi et al. 2021). Since these fungi degrade, it takes about 7–10 days for colour removal of the spent wash under laboratory conditions, whereas bioabsorption takes less time. (Savoca and Pace 2021).

### Use of fungi to decolourize spent wash

With a changing climate and reduced freshwater availability, there is considerable interest in wastewater treatment. The dark brown colour of spent wash is a major problem for distillery industries, and a significant amount of untreated spent wash is discharged into rivers and soil, causing pollution. As discussed above, the brown colour spent wash is toxic to aquatic life and harmful to soil health. Fungi can degrade various dyes, complex coloured compounds, and other xenobiotic compounds recalcitrant to biodegradation. This ability to adapt to severe environmental constraints, degrade pollutants, and treat industrial wastewater makes fungi an efficient tool for bioremediation purposes (Kharayat 2012; Mohana et al. 2008). In the past decade, fungi have been extensively studied for the bioremediation of distillery spent wash. Apart from reducing oxygen demand, fungi have also been reported to decolourize spent wash. Fungi are multicellular, produce extracellular enzymes, and increase the contact area, thus efficiently decolourizing spent wash (Kaushik and Malik 2009). There are several reports in which white-rot fungi such as *Phanerochaete chrysosporium*, *Trametes* (*Coriolus*) *versicolor*, *Bjerkandera adusta*, and the mutant *Flavodon flavus* have lignolytic properties. (Korniłłowicz-Kowalska and Rybczyńska-Tkaczyk 2021). Studies on the decolourization of spent wash using fungal culture have been done in two ways: either by focusing on the decolourization of spent wash or the breakdown of pure melanoidin by fungal strains. White wood-rot basidiomycetous fungi have been emphasized for the decolourization of dyes due to their high lignolytic activity (Revankar and Lele 2007). Laccase, lignin peroxidase, manganese peroxidase, and aryl alcohol oxidases have been known to degrade synthetic dyes (Kariminiaae-Hamedaani et al. 2007). Apart from dyes, these fungi have been reported to degrade melanoidin, the major colourant found in distillery spent wash (Pant and Adholeya 2007a, b, c). Members belonging to the genera *Coriolus, Fomitopsis, Irpex, Lenzites, Phanerochaete, Pleurotus, Poriba*, etc., are reported to decolourize the spent wash up to 85% (Table 2). Some of the previous studies have demonstrated the effective biodegradation of synthetic dyes such as Rhodamine B into environmentally non-toxic products. The researchers have shown that microbial and enzymatic mechanisms have been shown to help in dye breakdown through oxidative and reductive pathways, thus resulting in significant devolourization and detoxification of dyes. It highlights the potential of microbial systems in finding alternatives to conventional physicochemical dye treatment methods (Baldev et al. 2013).

**Table 2:** Fungi capable of decolourizing spent wash

S. no	Name	Comments	Colour removal %	References
1	Soil Fungi	High degree of decolourization in diluted spent wash	–	Kumar and Thankamani (2016)
2	*Aspergillus flavus,* *A. niger, A. terreus,* *Penicillium purpurogenum*	High degree of decolourization in diluted spent wash	65.10%	
3	*Alternaria gaisen,* *Penicillium pinophilum*	Immobilized using wheat straw and corn cob	47–50%	Pant and Adholeya (2007a, b, c)
4	*Monascus rhei,* *Tolypocladium inflatum,* *Penicillium chrysogenum,* *Aspergillus* sp., *Leptohaeria maculans,* *Coniothyrium fuckelli,* *Phaenerochaete chrysosporium*	High degree of decolourization in diluted spent wash	40–80%	Dahiya et al. (2001)
5	*Coriolus versicolor* Ps4a, *Coriolus hirsutus,* *Fomitopsis cytisina,* *Irpex lacteus* Ps8a, *Lenzites betulina* L5b, *Pleurotus ostreatus,* *Poria subacida*	23 genera, 30 strains of Basidiomycetes (white-rot and brow-rot) were tested for decolourization of distillery spent wash	17–80%	Aoshima et al. (1985)
6	*Coriolus hirsutus*	Inhibitory effect on melanoidin-degrading activity by nitrogen, the addition of Mn increased Mn peroxidase activity	–	Miyata et al. (2000)
7	*Coriolus* sp.	0.5% melanoidin medium was decolourized in two weeks	77%	Watanabe et al. (1982)
8	*Phaenerochaete chrysosporium*	Produces non-specific Lignolytic enzymes		Blondeau (1989)
9	*P.chrysosporium* NCIM 1073, *P. chrysosporium* NCIM 1106, *P. chrysosporium* NCIM 1197	Decolourization under submerged conditions	76–82%	Thakkar et al. (2006)
10	*P. chrysosporium*	COD reduction by 76.39%	78%	Singh et al. (2007)
11	*P. chrysosporium* *Geotrichum candidum* *C. versicolor* *Mycelia sterilia*	*P. chrysosporium* and *G. candidum* could grow in 50% of spent wash; Maximum decolourization was observed at 12.5% concentration of spent wash by *C. versicolor*	53%	Fitzgibbon et al. (1995)
12	*P. chryososporium,* *C. versicolor*	COD reduction at 6.25% diluted spent wash was 73% and 90%, respectively, for the two cultures	53–71%	Kumar et al. (1998)
13	*Mycelia sterilia*	BOD values were reduced by 90% over 15 days under non-sterile conditions	70%	Sirianuntapiboon et al. (1988)
14	*Flavodon flavus*	COD and phenolic concentrations were lowered by 50% over 8 days. Decolourizing activity was lost upon immobilization in alginate beads	80%	Raghukumar and Rivonkar (2001)
15	*C. versicolor,* *P. chrysosporium,* *Pleurotus pulomonaris,* *Funalia trogii*	Use of cotton stalk as a growth medium	–	Kahraman and Yesilada (2003)
16	*Pleurotus florida*	Decolourization of 50% post-methanated spent wash when immobilized on corn cob or wheat straw (28 days). Laccase production was enhanced	86%	Pant and Adholeya (2007a, b, c)
17	*Pleurotus sajor-caju*	Bioremediation of distillery effluent	–	Aragão et al. (2014)
18	*Ceriporiopsis subvermispora,*	Could lower BOD, COD, and total phenols from the wastewater and showed high laccase activity	–	Strong and Burgess (2007)
19	*Phaenerochaete chrysosporium, P. subceracea,* *P. sordida,* *Phlebia subochracea,* *Ph. subserialis,* *Pycnoporus coccineus,* *Py. cinnabarinus,* *Aleurodiscus disciformis,* *Bjerkandera adusta,* *Cerocorticium* sp., *Oligoporus caesius,* *Phlebia tremellosa,* *Schizophyllum commune,* *Trametes pubescens, T. hirsute,* *T. versicolor,* *Xylobolus spectabilis,* *X. subpileatus*	38 fungal strains (thermotolerant white rot fungi) were checked for laccase, manganese peroxide activity, and four strains of *P. coccineus* showed promising decolourization activity. The activity increased after immobilization on polyurethane film	–	Chairattanamanokorn et al. (2005)
20	*Geotrichum candidum*	showed stable peroxidase activity, which was prolonged with immobilization on polyurethane film	80%	Kim and Shoda (1999)
21	*Rhizoctonia sp.*	decolourization by sugar oxidases	87%	
22	*Penicillium pinophilum,* *Aspergillus flavus,* *A. gaisen, A. niger,* *Fusarium verticilloides,* *Pleurotus florida*	A consortium of fungal cultures showed COD removal by 64% over 14 days	61%	Pant and Adholeya (2010)
23	*Cladosporium cladosporiodes*	COD reduction by 62.5%	52%	Ravikumar et al. (2011)
24	*Fusarium flocciferum*	degradation of phenolic compounds	–	Mendonça et al. (2004)
25	*Neurospora intermedia*	biosorption of colourants by forming complexes between the colourant and ligands present on mycelia	–	Kaushik and Thakur (2013)
26	*Trichoderma viride,* *Trichoderma harzianum,* *Aspergillus niger,* *Alternaria alternate,* *Beauveria bassiana,* *Penicillium* sp., *Rhizopus* sp.	0.4% molasses medium showed the highest decolourization activity Decolourization activity varied among strains depending on the supplements used in the medium	40–53%	Seyis and Subasioglu (2009)
27	*Aspergillus oryzae*	Decolourization of 15% diluted spent was over 5 days, BOD & COD reduced by 63.9% and 48.7%	45–75%	Chavan et al. (2013)
28	*Aspergillus oryzae*	adsorption on dead biomass for 10% diluted spent wash	61%	Chavan et al. (2013)
29	*Aspergillus oryzae*	The medium used for the cultivation of the fungus affected the decolourizing activity	75%	Ohmomo et al. (1988)
30	*Aspergillus fumigatus*	COD and TOC reduction by 51% and 56%, respectively	60%	Ohmomo et al. (1987)
31	*Aspergillus niger*	Anaerobically treated spent wash was pre-treated with alum	97%	Shukla et al. (2014)
32	*Alternaria alternata,* *Aspergillus flavus,* *A. fumigatus, A. japnicus, A. ustus,* *A. versicolor,* *Cladosporium cladosporiodes,* *Curvularia lunata,* *Nigrospora spaherica,* *Penicillium oxygenum,* *P. purpurogenum,*	The fungal consortium was used to treat distillery spent wash and further subjected to secondary treatment using algal biomass immobilized in sodium alginate beads. *Sargassum wightii* showed maximum COD and BOD reduction, 65.8% and 71.05%, respectively	62%	Ravikumar et al. (2007)
33	*Aspergillus niger,* *A. flavus, Fusarium verticilloides,* *Pleurotus ostreatus*	The fungal cultures were immobilized on wheat straw and corn cobs	54–86%	Pant and Adholeya (2007a, b, c)
34	*Aspergillus oryzae*	A pre-treatment of biologically treated spent wash using calcium oxide/calcium hydroxide/ potassium permanganate/ bleach increased COD reduction (up to 94–96%) and colour removal	92–98%	Dhamankar and Patil (2001)
35	*Penicillium decumbens,* *Pencillium* sp., *A. niger, P. lignorum*	Separately, the four cultures achieved 50% BOD reduction. *P. decumbens* treated spent wash in an anaerobic reactor, lowering BOD values to 93%	40%	Jiménez et al. (2003)
36	*Aspergillus niveus*	Maximum decolourization and COD reduction (91.68%) were observed when bagasse was used for culturing the fungus	37%	Angayarkanni et al. (2003)
37	*Cunninghamella blakesleeana*	utilizing the distillery spent wash and synthetic melanoidin	1–10%	Kumar et al. (2012a, b)
38	*Candida tropicalis, Pediococcus acidilactici*	Spent wash decolourization	75%	Tiwari et al. (2014)
39	*Candida glabrata*	Four yeast cultures showed melanoidin degradation and manganese peroxidase activity, and decolourization was noted in 10% melanoidin wastewater	60%	Mahgoub et al. (2016)
40	*Saccharomyces cerevisiae,* *Candida intermedia,* *Hanseniaspora warum, Pichia membanaefaciens*	formed biofilm on a rotating biological reactor, lowering BOD by 96%	–	Coetzee et al. (2004)
41	*Issatchenika orientalis*	COD and BOD reduction by 77.4% and 80%, respectively, over 7 days when 2.5% glucose was externally supplied	91%	Tondee et al. (2008)
42	*Candida tropicalis*	Immobilized in alginate beads in consortium with *Pediococcus acidiacti*	85%	Tiwari and Gaur (2014)
43	*Hansuela fabianii,* *H. anomala*	Reduced the TOC of 1:5 diluted distillery effluent by 74.8%		Moriya et al. (1990)
44	*Trametes versicolor*	Decolourization in the form of pellets increased with an increase in inoculum	81%	Benito et al. (1997)
45	*Trametes* sp.	could decolourize 20% v/v molasses medium in seven days, with 61.7% COD reduction. Increased laccase production	73%	Gonzalez et al. (2000)
46	*Phanerochaete chrysosporium,* *Aspergillus niger,* *Pseudomonas aeruginosa*	The consortium, isolated from the spent wash contaminated site, showed a reduction in sulphites (96.8%), BOD (63.5%), and COD (59.4%)	87%	Pal and Vimala (2012)
47	*Aspergillus niger*	Pre-treatment using polyaluminium chloride	86%	Singh and Dikshit (2010)
48	*Flavodon flavus*	Immobilized using polyurethane foam and showed a reduction of polycyclic aromatic hydrocarbons	73%	Raghukumar et al. (2004)
49	*Citreomyces* sp.	almost 100% COD removal	76%	Sirianuntapiboon et al. (2004)
50	*Phanerochaete chrysosporium*	showed a correlation between the decolourization efficiency of spent wash due to lignin peroxidase and manganese peroxidase activity	75%	Vahabzadeh et al. (2004)
51	*Aspergillus niger*	Isolated from decaying wood	53%	Kale and Shinde (2020)
52	*Aspergillus brasiliensis*	Decolourization activity increased with increased starch (87.45%) and peptone (88.74%) concentration in the media	83%	Singh et al. (2020)
53	*Aspergillus, terreus*	Out of 37 fungal strains that were studied, the highest COD reduction was observed (77%) in two genera	43%	Chuppa-Tostain et al. (2020)
54	*Aspergillus niger,* *Aspergillus flavus,* *Cladosporium* spp., *Fusarium solani,* *Trichoderma viridi*	Decolourization of melanoidin was accompanied by a decrease in total suspended solids over a 7-day incubation period	–	Pathan (2022)

**Fig. 6:**
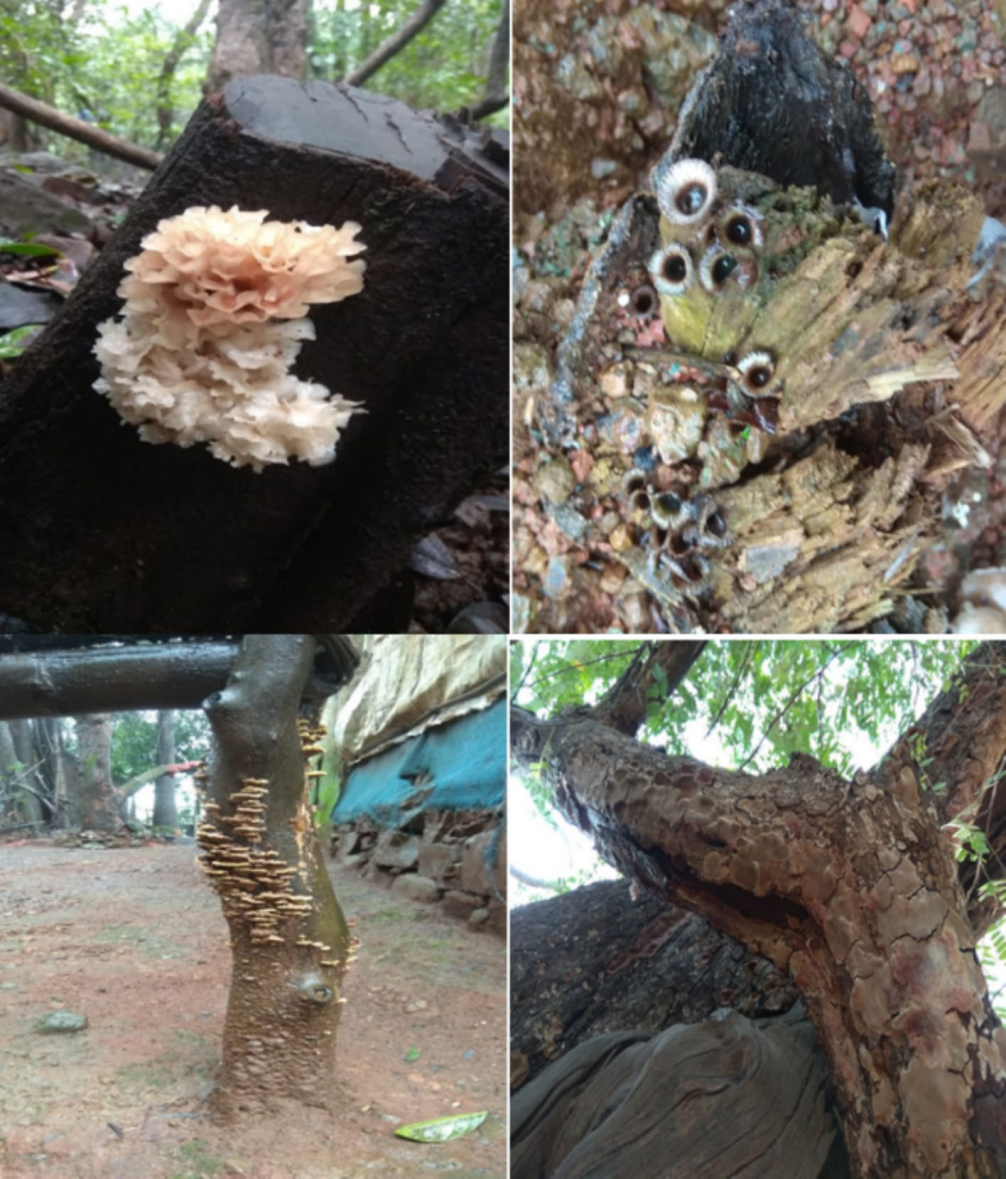
Wood-rotting fungi isolated from the Western Ghats region. The figure presents representative wood-rotting fungal species collected from the Western Ghats biodiversity hotspot, known for their ligninolytic enzymes and potential application in bioremediation and spent wash decolourization

However, not all wood-rotting fungi show the ability to degrade melanoidin and decolourize spent wash, as many of these fungi lack the enzymes and metabolic pathways for the degradation of melanoidin. The authors, while working on the screening of wood-rotting fungi, collected several fungi from forests of Western Ghats, India (Fig. 6). They observed that only 1–2% of the fungi showed positive activity to decolourize the spent wash. Amongst the wood-rotting fungi, *Phanerochaete chrysosporium* is the most studied fungus as it is known to decolourize spent wash (Thakkar et al. 2006), although interspecies and intraspecies variations exist. Sometimes these organisms are unable to utilize melanoidin as the sole carbon source, and the decolourization activity is attributed to the production of non-specific lignolytic enzymes. Some of the wood-rotting fungi that can decolourize spent wash are not able to break down pure melanoidin or produce laccase. Researchers have studied spent wash decolourization at 5–10%, since most fungi are unable to grow at higher concentrations. Moreover, not all distilleries produce spent wash of similar composition. There is a lot of variation in the spent wash composition due to the variety of sugarcane used, the method followed, and other parameters such as water quality (Asniah and Taufik 2025). Moreover, the ability of a fungal strain to decolourize the spent wash increases with the addition of inorganic nutrients. Mycelia sterilia has been reported to decolourize molasses spent wash up to 90% when supplemented with 2.5% glucose, 0.2% NaNO3, 0.1% KH2PO4, and 0.05% MgSO4, and the decolourization yield decreased in the absence of the above nutrients. Even though the strain showed good decolourization potential at low concentrations of spent wash and additional nutrients, under non-sterile conditions, the decolourization ability was reduced to 70%. In the absence of synthetic carbon and nitrogen sources, cotton stalk can be used as a growth medium to enhance the colour removal efficiency of *Coriolus versicolor*, *Phanerochaete Chrysosporium*, *Pleurotus pulmonarius*, and *Funalia trogii* (Kahraman and Yesilada 2003). Flavodon flavus could decolourize pigments in diluted spent wash by 80% throughout 8 days, but the strain lost its activity when F. flavus mycelia were immobilized in alginate beads (Raghukumar and Rivonkar 2001). Apart from *P*. *chrysosporium*, *Pleurotus florida* has shown a lot of potential for the decolourization of spent wash. It decolourized 50% post-methanated distillery spent wash up to 86% throughout 28 days (Pant and Adholeya 2007a, b, c).

### Enzymatic degradation of spent wash by fungi

A variety of enzymes have been known to be used for the treatment of wastewater from industries. Enzymes such as peroxidases, oxidoreductases, cellulolytic enzymes, cyanidase, proteases, and amylases come from diverse sources and are involved in degradation pathways (Kapoor 2018). Although an exact mechanism for the degradation of spent wash, in particular, melanoidins, is not known, enzymes such as lignin/manganese peroxidases, sugar oxidases, and laccases have been correlated with the decolourization of melanoidins, which can oxidize phenolic and non-phenolic moieties of the pigment. However, still more studies are required to confirm the enzymatic pathways. This can be done by identifying intermediates, final products, and mapping reaction networks, etc. Filtrates of *Trametes versicolor* have been reported to show a mineralizing effect on melanoidin, and the activity increased in the presence of Mn^2+^ and was related to a manganese-dependent enzyme, produced extracellularly. Anaerobically treated distillery spent wash can be used as a medium for laccase production, as was seen in the case of *Aspergillus heteromorphus* (Singh et al. 2010a, b). Enzymes are immobilized to minimize the loss of enzyme activity. Laccase enzyme immobilized on alumina and glass was able to reduce the colour of the baker’s yeast effluent by 68% after 24 h (Georgiou, et al. 2016). *Geotrichum candidum* was cultivated using molasses as a carbon source to effectively produce dye-decolourizing peroxidase (Lee et al. 2000). Manganese oxides have been reported for phenol oxidation properties and for colour removal activity from distillery spent wash by treating melanoidins (Rufián-Henares and Cueva 2009). Natural manganese oxides were reported to remove low molecular weight melanoidins preferentially. It is plausible that by having amine groups in the structure of melanoidins, the enzyme can dimerize the compound and precipitate it out of the wastewater (Arimi et al. 2015). Laccase activity was high in *Trametes* sp. when 20% distillery effluent supplemented with 1% glucose or fructose was used as media, with 60% decolourization. Extracellular lignolytic enzymes secreted by *P. florida* showed the highest decolourization of distillery spent wash (37%), attributed to high laccase concentration. However, maximum laccase and manganese peroxidase production was observed in *Fusarium verticillioides,* while maximum lignin peroxidase activity was noted in *Alternaria gaisen* (Pant and Adholeya 2009a, b). Enzymatic pretreatment of distillery spent wash has been known to reduce COD values (Sangave and Pandit 2006). The rate of aerobic oxidation increased two-fold when the spent wash was pretreated with cellulase enzyme due to the breakdown of complex molecules by enzymatic action (Sangave and Pandit 2006). In one of the studies, *Phanerochaete sordida, P. chrysosporium, Phlebia subochracea, P. subserialis,* and several strains of *Pycnoporus coccineus* showed high enzyme production, which increased when free mycelia were immobilized on polyurethane foam (Chairattanamanokorn et al. 2005). A fungal consortium was developed taking into account different enzymes produced by fungal cultures (*Penicillium pinophilum, Alternaria gaisen, Aspergillus flavus, A. niger, F. verticillioides,* and *P. florida*), and after 14 days, the undiluted distillery effluent showed colour and COD removal efficiency of 61.5% and 6.4% respectively (Pant and Adholeya 2010). *Pleurotus ostreatus* showed a high production of laccases, manganese-dependent peroxidases, and lignin peroxidases, which have been reported to aid spent wash decolourization (Pant and Adholeya 2007a, b, c).

### Factors affecting fungal decolourization

The decolourization of spent wash under laboratory conditions is subjected to various factors such as medium optimization factors, which include pH, C and N sources, metal cofactor(s), oxygen supply, agitation, substrate, etc. For a strain of *Aspergillus niveus*, the decolourization media was supplemented with glucose, KH_2_PO_4_, magnesium sulfate, and ammonium nitrate, while a strain of *Aspergillus niger* showed better activity in a media comprising KH_2_PO_4,_ magnesium sulfate, glycerol, and yeast extract (Dhamankar and Patil 2001; Angayarkanni et al. 2003; Naik et al. 2008). In *P. chrysosporium*, the presence of veratryl alcohol, humic acids, and melanoidins in different combinations showed a change in lignin peroxidase activity linked to the decolourization of spent wash. The acidic nature of the media caused a reduction of charges in humic acid, which could then be bound to fungal mycelia, leading to decolourization of the spent wash. *Cladosporium cladosporioides* preferred fructose and peptone as C and N-source, respectively, showing optimum growth at pH 5 and 35 °C (Ravikumar et al. 2013). A marine fungus, *Flavodon flavus,* showed better decolourization activity when seawater agar was used for growth, while *Fusarium flocciferum* showed better spent wash degradation activity in Yeast Nitrogen Base medium supplemented with ammonium sulfate (Raghukumar and Rivonkar 2001). In some fungi, continuous bubble reactors used to increase aeration showed better decolourization and COD removal. It was demonstrated that the use of potash alum, ferric chloride, and aluminum chloride as coagulants in a pre-treatment improved the decolourization activity of *A. niger* (Shukla et al. 2014). In a study involving *Neurospora intermedia,* a salt medium comprising of Na_2_HPO_4_, KH_2_PO_4_, MgSO_4_, Fe (CH_3_COO)_3_ NH_4_, Ca(NO_3_)_2_, NaNO_3_, gave the best results for adsorption studies (Kaushik and Thakur 2013). It becomes evident that the presence of a carbon and a nitrogen source in the medium facilitates the growth of the fungus and subsequent decolourization of the spent wash. The presence of a carbon source, such as glucose or sorbose, acts as a precursor to sugar oxidase enzymes, which help in the decolourization of spent wash. The enzyme releases active oxygen radicals, which can decolourize melanin (Watanabe et al. 1982). Aeration as a factor for spent wash treatment is debatable, as strains of *Pleurotus ostreatus, Aspergillus niger, A. flavus,* and *Fusarium verticillioides* showed decolourization in solid-state fermentation (Pant and Adholeya 2007c). The pH of the medium in this experiment was adjusted to a value of 6.5–7.0 to facilitate the growth of the fungus since the biomethanated spent wash has an alkaline pH. Upon decolourization, the pH of the medium turned acidic. The medium used for growing *A. oryzae* affected the melanoidin decolourizing activity (MDA) of the strain: the use of NaNO_3_ and mannitol, maltose, and glycerol increased MDA. The autoclaved mycelia showed consistent melanoidin adsorption, as did the resting mycelia. Organic nitrogen has been suggested to show an inhibitory effect on melanoidin degradation in *Coriolus hirsutus,* while the addition of manganese caused an increased production of Manganese peroxidase and subsequent increase in decolourization efficiency (Miyata et al. 2000). *Pleurotus ostreatus* immobilized using wheat straw and corn cob as a substrate showed 86% decolourization of 50% concentrated spent wash (Pant and Adholeya 2007b).

### Role of additional factors on fungal ability to decolourize

Several additional factors affect the decolourization potential of fungi, including solid substrates used to grow the mycelial mat, immobilization of the mycelia and enzymes, formation of beads, use of live or dead mycelia, etc. In one of the studies, fungal mycelia were immobilized using bagasse fibers, which showed a decolourization yield of 75.7% over 25 days. It was also noted that the enzyme activity of laccase, lignin peroxidase, and manganese peroxidase increased during this period. In another experiment, dead biomass of *Aspergillus oryzae* MTCC 7691 could absorb up to 61% of the colourants from 10% diluted spent wash (Chavan et al. 2013). The fungal consortium was used for the primary treatment of biomethanated spent wash. In another study, fungal biomass belonging to *Aspergillus niger* (two different strains), *A.* *flavus*, *Fusarium verticillioides,* and *Pleurotus ostreatus* were immobilized on wheat straw and corn cob. *P. ostreatus* showed a maximum decolourization of distillery spent wash up to 86.33% throughout 28 days. When *Candida tropicalis* was immobilized in a 2% sodium alginate bead in consortium with *Pediococcus acidilacti,* the decolourization of melanoidin reached up to 85%. The beads could be reused for eighteen cycles (Tiwari et al. 2014). *Trametes versicolor,* used in the form of pellets, was reported for the decolourization of molasses spent wash by 81%. An increment in the initial inoculum concentration led to an increase in decolourization, where sucrose and monopotassium phosphate served as sources of additional nutrients. Another species belonging to *Trametes* sp. I-62 could decolourize 20% (v/v) of molasses medium over seven days. The decolourization yield was 73.3%. A 35-fold increase in laccase production was noted, and a pyrolysis/ gas chromatography/ mass spectrometric analysis confirmed the degradation of melanoidins by the fungus (Gonzalez et al. 2000). A consortium of *Phanerochaete chrysosporium* with *Pseudomonas aeruginosa* and *Aspergillus niger,* respectively, isolated from distillery effluent contaminated soil, showed decolourization activity of 87.8% throughout 15 d, which was higher than when the organisms were used solo (Pal and Vimala 2012). A marine isolate of *Flavodon flavus* decolourized 10% distillery spent wash by 73% when immobilized with polyurethane foam. The presence of polycyclic aromatic hydrocarbons (PAH) in the effluent contributed to its toxicity, and the immobilized fungus could decrease the PAH content by 68% over five days (Raghukumar et al. 2004). A yeast strain isolated from fruit samples, identified as *Citreomyces* sp., was reported to reduce the oxygen demand of the spent wash but did not affect the colour (Sirianuntapiboon et al. 2004). *Phanerochaete chrysosporium* in the presence of higher concentrations of manganese and urea showed decreased colour removal and lignin peroxidase activity. On a 10X diluted media, the fungus could decolourize the spent wash up to 75% over five days. The study showed a correlation between the production of lignin peroxidase and manganese peroxidase with the decolourization efficiency of the culture (Vahabzadeh et al. 2004).

### Fungal decolourization mechanisms and toxicity of breakdown products

Although the exact mechanism of melanoidin degradation is not known, researchers have tried to propose possible mechanisms based on their preliminary laboratory experiments. These need more validation and detailed studies. One of the mechanisms proposed is the degradation of melanoidin by extracellular laccase. Fungi have been reported to degrade melanoidin by producing laccases and other lignolytic enzymes. These fungi can metabolize melanoidins as a source of carbon and nitrogen (Miyata et al. 2000; González et al. 2008). However, in-depth molecular mechanisms and kinetics are unknown. The mechanism of melanoidin degradation by laccase enzymes was studied in a *Megasporia* sp. Strain (Toomsan et al. 2020). The highest degradation efficiencies were 48.00% and 44.60% for purified and crude laccases, respectively, and fit the Michaelis–Menten model of enzyme kinetics. Another mechanism proposed for the decolourization of melanoidins speaks of the absorption of the compound, which exists in the cell membrane and cytoplasm in the form of a complex and is then decolourized by intracellular enzymes, mainly sugar oxidases, as had been described previously. The mechanism has been reported in *Rhizoctonia* sp., which could decolourize molasses medium by 87.5% and synthetic melanoidin by 84.5% and *Aspergillus* sp., which could decolourize up to 69–75%. While developing a biotechnological process, the breakdown products from the reaction need to be focused on. These products should ideally be non-toxic, or at least less toxic than the starting product. There is a gap in our knowledge about the breakdown products of melanoidin using wood-rotting fungi. Studying these will help us to understand the mechanism involved and the products produced after the breakdown of melanoidin.

## Other microbial methods

At the present time, the integration of different capacities of microbes and other organisms is an important aspect of the development of biotechnology. Keeping this in mind, the potential of bacteria, cyanobacteria, and plants is discussed below for their colour removal ability, and various studies are reviewed. Most bacteria do not show very high decolourization potential, but some have shown promising results. *Bacillus licheniformis*, *Bacillus* sp., and *Alkaligens* sp. showed decolourization of synthetic and natural melanoidin in the range of 52–66% when used anexically. In the consortium, the organisms could decolourize synthetic and natural melanoidins by 73.79% and 69.83%, respectively (Bharagava et al. 2009). Bacterial strains have been reported to decolourize the spent wash up to 68% viz., *Lactobacillus Plantarum*, *Pseudomonas aeruginosa*, *Stenotrophomonas maltophili,* and *Proteus mirabilis* (Tondee and Sirianuntapiboon 2008; Mohana et al. 2007). The efficacy of consortia has been variable17.5% (*Klebsiella oxytoca*, *Serratia marcescens,* and *Citrobacter* sp.) to 75% (*Proteus mirabilis*, *Bacillus* sp., *Roultella planticola* and *Enterobacter sakazakii*) (Jiranuntipon et al. 2008; Yadav and Chandra 2012). Acetogenic bacteria have also been reported for the decolourization of distillery spent wash, the highest yield being reported for strain BP103 as 76.4% over five days in a modified spent wash medium containing 3% glucose.

However, while working with a three-stage bioreactor, a consortium of bacteria and fungi was used to treat distillery spent wash. The fungal species (*Emericella nidulans* and *Neurospora intermedia*) could decolourize the wastewater, while the bacteria (*Bacillus* sp.) reduced the oxygen demand values. After 30 h, the treated effluent showed an 82% colour reduction (Kaushik et al. 2010). A consortium of *Pediococcus acidilacti* and *Candida tropicalis*, both isolated from distillery effluent-contaminated soil, was developed and showed 82.1% decolourization of spent wash within 24 h (Tiwari et al. 2014). A consortium of aerobic bacteria consisting of *Klebsiella pneumoniae, Salmonella enterica, Enterobacter aerogenes,* and *Enterobacter cloacae* showed degradation of distillery spent wash by 81% through co-metabolism when glucose and peptone were used as nutrient sources. Laccase and manganese peroxidase activity were detected in the supernatant, suggestive of the role they played in decolourization (Chandra et al. 2018). A consortium of *Proteus mirabilis, Bacillus* sp., *Raoultella planticola,* and *Enterobacter sakazakii* decolourized spent wash up to 76% within 192 h. Lignolytic enzymes (manganese peroxidase and laccase) were associated with colour removal activity (Yadav and Chandra 2013).

### Phycoremediation

Several researchers demonstrated the use of algal biomass for remediation for bioadsorption of nutrients (N, P) and heavy metals, such as Cu, Cd, Zn, Hg, Pb, etc., or biotransformation of xenobiotics using algal biomass is known as phycoremediation (Bezuneh 2016; Tripathi et al. 2019; Knutsson et al. 2016). The high versatility of their metabolic mechanisms presents cyanobacteria and microalgae as a lucrative option for bioremediation (Amores-Sanchez et al. 2015). The simultaneous biomass production can be exploited as protein supplements, food additives, bioenergy resources, or in pharmaceutical and cosmetic industries (Renuka et al. 2015). Mixotrophic algae, such as *Botryococcus braunii*, are known to remove inorganic and organic salts from wastewater, which can be used for biomass production in the algae, simultaneously treating the wastewater (Kong 2012). Phytoremediation is considered a possible option in existing treatment plants using algae such as *Spirulina* sp., *Oscillatoria* sp., etc. Anaerobically digested spent wash was subjected to treatment by microalgae, which reduced the nutrient load for the water to be treated in reverse osmosis plants (Krishnamoorthy et al. 2019). Distillery effluent treated by microalga, such as *Chroococcus minutus* can be used for the cultivation of crops such as *Cicer arietinum* (Murugesan et al. 2017). Apart from nutrient utilization, microalgae show melanoidin decolourization as well. *Phormidium*, *Spirulina,* and *Synechococcus* have been reported for the decolourization of melanoidin. *Oscillatoria boryana* BDU 92181 could decolourize 0.1% (w/v) pure melanoidin and 5% (v/v) distillery effluent up to 60% throughout 60 days. The mechanism of melanoidin degradation has been proposed to be the release of hydroxyl ions, free O_2,_ and hydrogen peroxide during photosynthesis (Francisca Kalavathi et al. 2001). A consortium of *Oscillatoria*, *Lyngbya,* and *Synechocystis* showed decolourization of spent wash up to 98%. Individually, the organisms could show decolourization up to 96%, 81%, and 26%, respectively (Patel et al. 2001). The mechanism of decolourization was speculated to be the absorption and degradation of organic compounds. A lab-scale semi-batch culture was used to study the bioremediation of distillery effluent by *Chlorella sorokiniana*. A stable microalga-bacteria consortium was developed, which could lower spent wash pollutants over four days (Solovchenko et al. 2014). *Chlorella vulgaris* could decolourize 10% of spent wash up to 52% and reduce other pollutants in treated spent wash used to grow *Lemna miniscula*. However, the macrophyte could not help in the removal of nutrients (Valderrama et al. 2002). In a two-stage sequential treatment, *Vetiveria zizanioides* and *Phragmites kharka* were employed to reduce the total nitrogen content of the distillery spent wash, which was reduced by 84%. The treated spent wash was then subjected to colour removal by *Pleurotus florida* (86.33%) and *Aspergillus flavus* (74.67%) (Pant and Adholeya 2009a, b). The effluent treatment potential of *Cladosporium cladosporioides* and *Phormidium valderianum* in a two-stage biological sequestration was studied. Fungal culture decolourized the spent wash by 68.5% which increased in the second stage to 92.7% when treated with cyanobacteria (Ravikumar and Karthik 2015). Several other cyanobacteria have been tested for the decolourization of the spent wash. However, most of the cyanobacteria play a role in reducing the oxygen demand and other pollutants, but have little role in the colour removal of spent wash.

## Non-biological treatment of distillery spent wash

Apart from the biological method, it is also important to briefly discuss non-biological methods, which are important for process development. Physico-chemical treatments have been proposed for the decolourization of spent wash at either primary or tertiary stages for wastewater treatment (Kharayat 2012). Microfiltration has also been tested, which could efficiently remove 42.7% colour and other contaminants, increasing Reverse Osmosis (RO) performance and water recovery (Sharma and Joshi 2016). Activated carbon is efficient in adsorbing coloured components from distillery spent wash, such as tannins and melanoidins (Figaro et al. 2006). Steam activation of carbon prepared from bagasse, sawdust, bagasse fly ash, wood ash, and rice husk ash was reported to 50% decolourize the spent wash. However, the commercially available activated carbon showed 80% decolourization (Satyawali and Balakrishnan 2008). Spent wash pretreated in an anaerobic floating bed baffled reactor was subjected to further treatment using activated bagasse, which showed a colour removal efficiency of 68.9% and melanoidin reduction by 40% when bagasse was chemically modified using 2-diethylaminoethylchloride (DEAE-bagasse) and 3-chloro-2-hydroxypropyltrimethylammonium chloride (CHPTAC-bagasse) (Lakshmikanth and Virupakshi 2012; Mane et al. 2006). It was suggested that the mechanism could be via both ion exchange and chemical sorption. Activated carbon prepared from *Piper nigrum* stem showed decolourization of distillery spent wash up to 75% with a considerable amount of melanoidins’ removal when used in a continuous fixed bed column (Arulmathi and Elangovan 2016). Adsorbents fabricated from charcoal fly ash and clay were used for the treatment of molasses (10% solution), and a melanoidin removal efficiency of 82% was observed (Ramezani et al. 2011). Advanced oxidation processes for the degradation of various organic contaminants have been studied extensively, and the use of solar photocatalysis for the treatment of wastewater is well documented in the literature. Titanium dioxide plates were used for decolourization from biomethanated spent wash and showed positive results when 100 times diluted spent wash was used (Akbarzadeh et al. 2016). Vanadium-doped TiO_2_ nanoparticles were prepared using a sol-gel method and showed 65% colour removal of spent wash in 5 h when irradiated with natural sunlight (Takle et al. 2018). Advanced oxidation processes, when combined with Ozone treatment and the Photo-Fenton process, reduce colour. A combination of O_3_/UV/Fe_2_+/H_2_O_2_ showed 100% colour reduction efficiency, which can be attributed to the fact that at higher concentrations of H2O2, scavenging of -OH radicals increases (Asaithambi et al. 2015). Nano-photocatalysts were prepared using aluminum oxide and kaolin clay, and 80% colour removal of spent wash was achieved (David et al. 2015a). A possible mechanism of decolourization of melanoidins is oxidative decomposition. Treatment with H_2_O_2_ and ozone was shown to remove colour by 65%. Combining UV and H_2_O_2_ with ozonization reduced colour by 88% (Kolte et al. 2014). When vinasse was pre-treated by ozonation, almost 50% of phenols were reduced to simpler forms (Siles et al. 2011). Chemical coagulants such as aluminum sulfate, calcium sulfate, and ferric sulfate have been used for wastewater treatment. When used with *Moringa oleifera* seed extract, the colour removal efficiency of the coagulant (ferric sulphate) was increased up to 96.5% (David et al. 2016). Ferric chloride, when used as a coagulant, decolourized the effluent up to 90% at pH 5. Simultaneously, electro-flocculation with a current intensity of 0.5A was performed and showed up to 90% colour removal with real untreated wastewater (Liakos and Lazaridis 2014). Electro-coagulation is an effective process for the removal of colourants from distillery spent wash (79% colour removal efficiency) as compared to Fenton (66%) and electro-fenton (44%) processes (David et al. 2015a, b, c). Decolourization of distillery spent wash using a biopolymer synthesized by *Pseudomonas aeruginosa* isolated from tannery effluent was also studied (David et al. 2015a, b, c). Electrocoagulation of 17.5% diluted spent wash with a current density of 31 mA/cm^2^ for four hours showed a colour reduction of 93.5%, which was close to the predicted value of 95% by response surface methodology (Krishna Prasad et al. 2008). In a batch reactor, electrocoagulation using aluminum and iron electrodes, as well as chemical coagulation using alum and lime, was studied. Al-Al electrodes showed maximum colour removal (96.06%) at pH 8 (Wagh and Nemade 2015). Ozone-assisted electrocoagulation using aluminum plates was carried out as a post-treatment for anaerobically digested distillery spent wash. The process achieved 92% decolourization at a current density of 9.75 Acm^−2^ (Wagh and Nemade 2017). Ionizing radiations at a dose of 4.5 kGy and 14 kGy showed chromaticity reduction of 27.5% and 75% in a dialyzed sample of distillery spent wash. The biodegradability of the effluent increases with a decrease in pH, and organic acids such as formic acid, oxalic acid, and glycolic acid are formed with an increasing dose of radiation. An anaerobic up-flow fixed bed reactor with an annular photo-catalytic reactor was used to study the effect of anaerobic digestion and UV degradation of spent wash and raw molasses wastewater, and achieved 88% colour removal when UV photo-degradation was used post anaerobic digestion. UV photo-degradation alone showed 54% and 69% colour removal from distillery effluent and molasses wastewater, respectively, whereas anaerobic digestion showed 51% colour removal from spent wash (Apollo et al. 2013).

## Recent advances (Genome sequencing and metagenomic studies)

Recently, a lot of work on uncultured microbial studies has been undertaken to understand and recognize the structure and ecology of an anaerobic digester by studying the spent wash released from it. Biomethanated spent wash has a dominant bacterial population belonging to *Bacillus* and *Stenotrophomonas,* belonging to the phyla *Firmicutes* and *Gamma-Proteobacteria*, as was revealed by restriction fragment length polymorphism. The presence of these species indicated their adaptability to acidic environments and rendered them pioneer taxa for in situ remediation of the spent wash. The presence of methanogenic archaea was also detected (Chandra and Kumar 2017a, b). A phylochip analysis revealed *Firmicutes* as the dominant phylum associated with sugarcane processing plants (Sharmin et al. 2013). Metagenomic studies revealed a difference in the microflora associated with Microbial Fuel Cells (MFC) treating anaerobic sludge from two distinct climatic zones. Although *Proteobacteria, Bacteriodetes,* and *Firmicutes* formed the dominant core of the MFC population, the initial inoculum community from the United Kingdom (UK) sample constituted *Bacteriodetes* in abundance, followed by *Firmicutes, Proteobacteria,* and *Actinobacteria*; while the initial inoculum community from Japan (JP) had a greater diversity with *Proteobacteria* in abundance and *Archaea* present in significant amounts. Both communities underwent structural changes throughout the MFC operation. Proportions of *Proteobacteria, Firmicutes,* and *Actinobacteria* increased in UK-MFC, although *Bacteroidetes remained the dominant phylum*. In JP-MFC, *Bacteroidetes, Firmicutes,* and *Archaea* increased in abundance (Kiseleva et al. 2015). The community structure of Internal Circulation reactors treating baggage wastewater was studied under two conditions: with and without the addition of molasses wastewater. The most abundant phylum in both reactors was *Firmicutes, followed by Bacteroidetes* and *Proteobacteria*. Although the pattern remained the same, the abundance of the organisms varied in the two reactors. The presence of bacteria such as *Clostridiales, Bacteroidales, Desulforomondales, Syntrophobacterales, Desulfovibrionales, Spirochaetales, Actinomycetales, and Nitrospirales* is associated with the degradation of components present in molasses wastewater, such as the oxidation of unsaturated and saturated fatty acids, the degradation of amino acids, the reduction of sulfates to hydrogen sulfide, etc. The archaeal community of the reactors had members belonging to the genus *Methanosarcina, Methanosaeta,* and *Methanolinea* (Shen et al. 2013).

The recent advances in metagenomics studies are a boon to research on the decolourization of spent wash. The metagenomics of biomethanated spent wash can help to understand the structure of microbial communities, succession activities inside the anaerobic digester during the whole process, and to some extent, the functions of these communities. In turn, it is helpful to know the competition that the selected fungi will have to face while treating the spent wash. It is an essential factor when a laboratory technology has to be tested at a large scale and performed successfully. We know that it is difficult to cultivate all the microbes living inside the anaerobic digester, which hampers our ability to completely understand it. It creates hurdles in the development of a successful biotechnological process. As discussed above, studies have revealed that the anaerobic digester is dominated by a bacterial population; it is better to understand the interaction between the selected fungi and the bacterial population and factors such as pH, temperature, secondary metabolites, etc., that might affect the outcome of a technology when put in the field. Similarly, genome sequencing of the selected fungal strain for the development of a biotechnological process is an important factor in understanding the physiology, metabolism, and behavior of the fungi. It is believed that Whole Genome Sequencing will help to know the pathways present in the selected fungi and their possible breakdown products (Lodi et al. 2025). This will help determine the toxicity of the final product, which will be released along with the treated spent wash. It may also help in understanding the potential of the selected fungi to address the physicochemical conditions of the biomethane spent wash and in predicting its tentative performance in the field. Although it remains to be substantiated by practical laboratory experiments.

## Conclusion

**Fig. 7:**
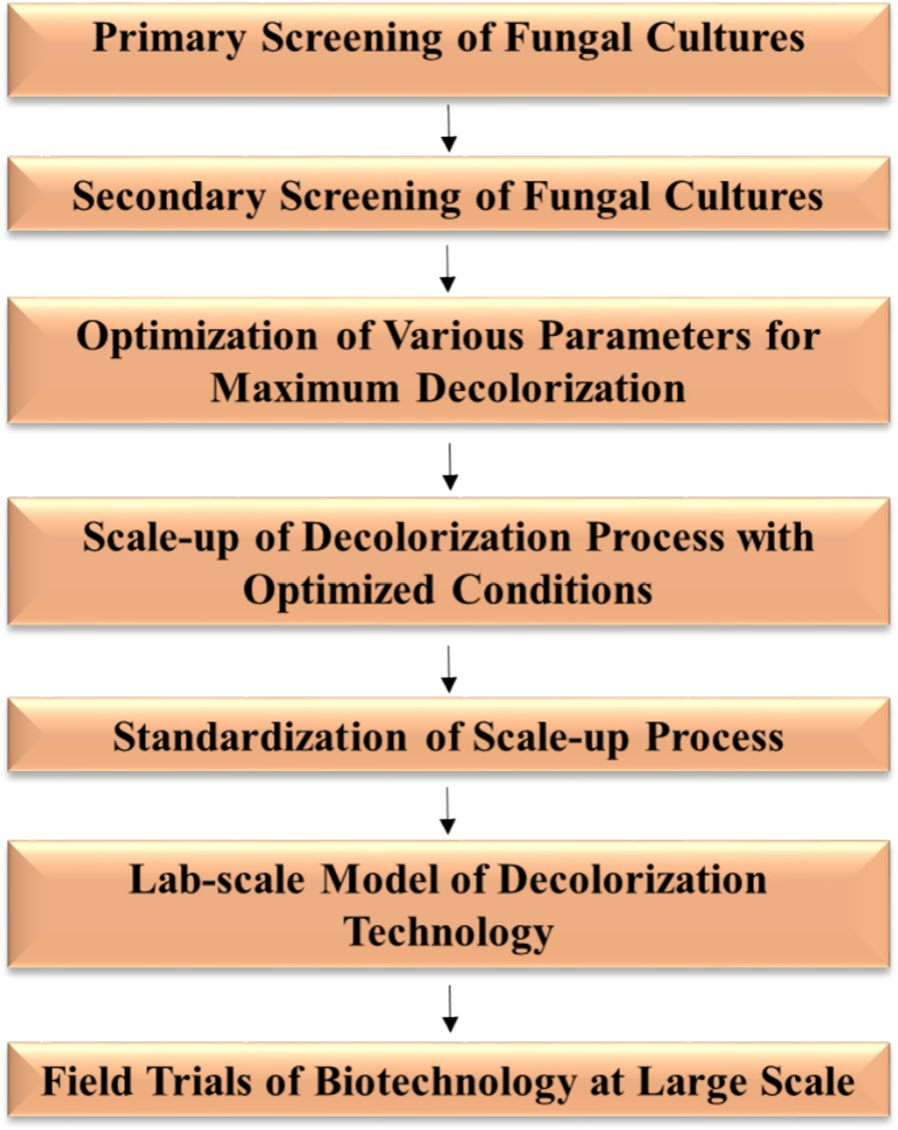
Schematic representation of the biotechnology-based process for spent wash decolourization. The above diagram illustrates the sequential steps involved in fungal-based decolourization of distillery spent wash, including fungal inoculation, enzyme action, breakdown of melanoidin, and reduction in colour intensity

Distillery spent wash is a highly persistent and polluting industrial effluent that severely impacts water bodies and soil health. Over the past two decades, numerous biological and non-biological methods have been explored for its treatment, with fungal-based approaches showing exceptional promise in laboratory studies (Ratna et al. 2021). Notably, wood-decaying fungi such as *Phanerochaete chrysosporium*, *Pleurotus ostreatus*, *Trametes versicolor*, and *Flavodon flavus* have demonstrated significant potential for decolourizing spent wash through their ligninolytic enzyme systems. The key to advancing fungal biotechnologies lies in targeted isolation and systematic screening of diverse fungal strains, followed by selecting the most efficient ones. However, two major limitations hinder the direct application of fungal treatment to biomethanated spent wash: the need for additional carbon sources and the dilution of the effluent required to support fungal growth (Naik et al. 2008). Similarly, physico-chemical methods such as coagulation-flocculation, adsorption, electrocoagulation, and advanced oxidation have achieved substantial colour and COD removal but often suffer from high energy demand, reagent use, and sludge generation. Integrating fungal treatment with optimized physico-chemical pre-treatment could overcome these challenges and lead to a scalable, eco-friendly solution for the development of efficient spentwash treatment technology (Fig. 7).

Another important point is the drawbacks of current treatment methods like lagooning, land application, composting, and incineration. Lagoons and uncontrolled land application only dilute or redistribute the pigment and toxicity of spentwash. Composting with press mud stabilizes organic matter, but melanoidins and colour are not reduced. (Umair Hassan et al. 2021). Long-term use of pressmud damages soil health and induces phytotoxicity. Therefore, its direct use in agricultural fields is generally not recommended. However, due to a lack of appropriate policies, many distilleries discharge waste into nearby rivers during the rainy season, when the water becomes muddy. Even spent wash stored in lagoons is disposed of in a similar way, causing damage to nearby agricultural fields due to seepage from lagoons. Therefore, an effective discharge and management policy is imperative to prevent further environmental damage.

One of the most pressing hurdles is translating successful lab-scale results into industrial-scale operations. Moving from flask-level (1 L) to distillery-level (40,000 L) is a complex process that must be approached in phased, proportionate steps. This up‑scaling must explicitly address hydrodynamics, oxygen transfer, mixing, and process control to maintain fungal activity and enzyme production under variable effluent characteristics (Hiremath and Joshi 2022). However, studies are still required to scale up the technology to 40,000–50,000 L of spent wash. Another key challenge is maintaining fungal activity in undiluted or minimally diluted distillery spent wash. In addition, a continuous culture of the desired fungal strain and maintaining a short retention time is challenging for scale-up purposes. Long‑term pilot and full‑scale demonstrations integrating process control, reactor engineering, and techno‑economic assessment still remain limited. Additionally, metagenomic and physicochemical analyses of anaerobic digesters can provide vital insights into the microbial interactions and environmental stressors that fungi may face during large-scale application. Fungal strains typically thrive in 10–15% diluted spent wash, with some capable of tolerating up to 50%. Therefore, integrating physical or chemical treatments, such as UV irradiation, hydrogen peroxide, or activated charcoal, could reduce melanoidin concentrations, enabling fungi to function more effectively with less dilution. This would significantly lower water usage during treatment, making the process more sustainable.

The ecological risks of biomethanated (post‑methanated) DSW still contain colour, recalcitrant organics, and organo‑metallic pollutants capable of causing chronic toxicity to fish and crops at low dilutions. Long‑term risk management requires continuous monitoring of colour, COD, priority pollutants, and ecotoxicity. Post‑treatment discharge or reuse for irrigation needs stringent monitoring to avoid soil salinization and groundwater contamination.

Moreover, understanding the breakdown products of melanoidin is essential to ensure that the end-products of decolourization are environmentally safe. Even if the treated water is not potable, it can still be repurposed for agricultural or industrial applications, easing the pressure on freshwater resources. To ensure the reliability of fungal treatment technologies, rigorous industrial-scale trials are imperative. Since the composition of spent wash can vary significantly across distilleries and raw material types (e.g., sugarcane variety), it is crucial to evaluate fungal strains on diverse effluents and, if necessary, customize treatments to local conditions. In this context, techno‑economic and life‑cycle assessments are needed to benchmark fungal‑based hybrids against existing options such as biomethanation, incineration, composting, and zero‑liquid‑discharge schemes (Borges Silva and Kardos 2024). Developing a classification system for spent wash based on its origin and processing method would further aid in matching the right fungal strain with the appropriate effluent type (Fig. 7). An integrated approach combining physico-chemical and microbial processes is preferred for distillery spent wash treatment. A laboratory-scale technology developed by the authors integrates all three processes, achieving about 85–90% melanoidin degradation, as confirmed using high proficiency liquid chromatography (HPLC). Studies are in process to identify and understand the toxicity of the new breakdown product.

In conclusion, this is the right time to recognize the severity of distillery waste pollution and take decisive steps towards sustainable solutions. Fungal bioremediation, especially when integrated with complementary physical and chemical technologies, holds great promise. Integrating anaerobic digestion, microbial enzymatic interventions and the valorization of treated effluents and by-products can turn distillery spent wash into a resource. Biomethanation can convert organic load into biogas. Moreover, the fungal treatment can detoxify and de-colourize the post-methanated effluent, enabling its reuse in irrigation or other uses of processed water, thus reducing the demand for fresh water. Co-management of distillery spent wash with other residues, such as bagasse, press mud, and biochar, thus limiting environmental damage. Positioning distillery spent wash as both a treatment challenge and a resource through biogas, bio‑nutrient recycling, and value‑added products aligns fungal processes with circular‑economy goals and emerging regulatory requirements for zero‑liquid discharge. Although stringent discharge standards for distillery effluents exist in several countries, enforcement remains inconsistent, and regulations largely focus on bulk parameters (COD, BOD, TDS) rather than specific recalcitrant organics and melanoidin-associated colour, indicating policy gaps for safe land applications (Patel et al. 2023). Wholistic treatment of the spent wash make it reusable, it would represent a significant advancement in wastewater management and environmental conservation.

## Data Availability

Not applicable.
